# Activation of Grm1 expression by mutated BRaf (V600E) *in vitro* and *in vivo*

**DOI:** 10.18632/oncotarget.23637

**Published:** 2017-12-23

**Authors:** Ho-Chung Chen, Jairo Sierra, Lumeng Jenny Yu, Robert Cerchio, Brian A. Wall, James Goydos, Suzie Chen

**Affiliations:** ^1^ Susan Lehman Cullman Laboratory for Cancer Research, Ernest Mario School of Pharmacy, Rutgers, The State University of New Jersey, Piscataway 08854, NJ, USA; ^2^ Rutgers-GSBS at Robert Wood Johnson Medical School, Piscataway 08854, NJ, USA; ^3^ Pharmacology and Toxicology Graduate Program, Rutgers University, Piscataway 08854, NJ, USA; ^4^ Global Product Safety, Colgate-Palmolive Company, Piscataway 08854, NJ, USA; ^5^ Rutgers Cancer Institute of New Jersey, New Brunswick 08903, NJ, USA

**Keywords:** mutated BRaf (V600E), Grm1, melanoma, senescence, p15

## Abstract

Our laboratory previously showed that ectopic expression of Grm1 is sufficient to induce spontaneous melanoma formation with 100% penetrance in transgenic mouse model, TG-3, which harbors wild-type BRaf. Studies identified Grm1 expression in human melanoma cell lines and primary to secondary metastatic melanoma biopsies having wild-type or mutated BRaf, but not in normal melanocytes or benign nevi. Grm1 expression was detected in tissues from mice genetically engineered with inducible melanocyte-specific BRaf^V600E^. Additionally, stable clones derived from introduction of exogenous BRaf^V600E^ in mouse melanocytes also showed Grm1 expression, which was not detected in the parental or empty vector-derived cells, suggesting that expression of BRaf^V600E^ could activate Grm1 expression. Despite aberrant Grm1 expression in the inducible, melanocyte-specific BRaf^V600E^ mice, no tumors formed. However, in older mice, the melanocytes underwent senescence, as demonstrated previously by others. It was proposed that upregulated p15 and TGFβ contributed to the senescence phenotype. In contrast, in older TG-3 mice the levels of p15 and TGFβ remained the same or lower. Taken together, these results suggest the temporal regulation on the expression of “oncogenes” such as Grm1 or BRaf^V600E^ is critical in the future fate of the cells. If BRaf^V600E^ is turned on first, Grm1 expression can be induced, but this is not sufficient to result in development of melanoma; the cells undergo senescence. In contrast, if ectopic expression of Grm1 is turned on first, then regardless of wild-type or mutated BRaf in the melanocytes melanoma development is the consequence.

## INTRODUCTION

Melanoma is the most dangerous form of skin cancer. Estimated new cases in 2017 exceed 76,000 and projected deaths this year exceed 10,000 [1]. Several risk factors have been identified, including UV radiation exposure, family history, and immunosuppression [2, 3]. It remains unclear if the majority of melanoma develops from benign nevi, groups of melanocytes that have undergone several rounds of proliferation mediated by an oncogene that were insufficient to bring about full transformation and tumorigenesis before undergoing oncogene-induced senescence. If a second-hit mutation occurs, the nevus may progress to melanoma.

Three major pathways have been identified as critical players in melanomagenesis: the mitogen-activated protein kinase (MAPK), brought about by activating mutations in RAS or RAF, and KIT, a receptor tyrosine kinase, involved in cell proliferation; PI3K/AKT, due to loss of function of PTEN or dysregulation of AKT; and p16/INK4a and p14, due to mutations in cyclin-dependent kinase inhibitor 2A (CDKN2A [2, 4, 5]. Many of the therapeutic strategies to treat melanoma are based on targeting mutated proteins in these various dysregulated pathways. Checkpoint blocking antibodies such as Ipilimumab, pembrolizumab/nivolumab, vemurafenib and trametinib as well as specific inhibitors of mutated BRaf (BRaf^V600E^) and MEK, have emerged as promising new therapeutic approaches [6–9]. However, tumor cells frequently gain resistance to these drug treatments and patients relapse within months [10–12]. These outcomes might be due to the heterogeneity of melanoma [5, 13] or crosstalk between multiple signaling pathways, which is Context-dependent [14]. For example, when growth factor levels are high, AKT phosphorylates BRaf at two sites to down-regulate its catalytic activity, which subsequently lowers MAPK pathway activity, while under low growth factor levels PI3K can induce RAF and MEK expression [14].

Our lab previously showed that ectopic expression of metabotropic glutamate receptor 1 (Grm1) is sufficient to induce spontaneous melanoma formation with 100% penetrance in a transgenic mouse model [15, 16]. Subsequent studies identified Grm1 expression in 80% of human melanoma cell lines and 65% of primary to secondary metastatic melanoma biopsies, but no Grm1 was detected in normal melanocytes or benign nevi [15, 17, 18]. Stable Grm1-expressing mouse melanocytic clones, MASS clones, were established to elucidate the mechanism underlying Grm1-mediated signaling pathways in melanoma. The MAPK pathway showed constitutive activation; in contrast, the PI3K/AKT pathway was only stimulated in the presence of quisquilate, an agonist of Grm1, insulin-like growth factor (IGF-1), or in in vivo MASS-tumors [18–21]. Further investigation demonstrated transactivation of insulin-like growth factor-1 receptor (IGF-1R) by Grm1, another example of crosstalk between G-protein-couple receptors (GPCRs) such as Grm1 and receptor tyrosine kinases (RTKs) such as IGF-1R [21–23]. Cultured cells derived from metastatic human melanoma such as C8161 and UACC903 show elevated basal levels of activated phospho-ERK [pERK] and phospho-AKT [pAKT] compared to Grm1-negative human primary melanoma line, C81-61, and other normal human melanocytic cell lines [4].

The most common mutation in BRaf, is a single nucleotide mutation resulting in substitution of glutamic acid for valine (BRaf^V600E^ .: nucleotide 1799 T>A) [24–26]. BRaf^V600E^ is observed in more than 60% of melanomas, and leads to hyperactivation of the MAPK pathway [24–26]. Earlier studies have shown that mutated BRaf^V600E^ alone is not sufficient to transform melanocytes into tumorigenic cells; rather it triggers cells to undergo senescence [27, 28]. Further genetic mutations in PTEN or p16/INK4a are necessary for these mutated BRaf-induced senescent cells to initiate re-entrance into the cell cycle to progress to aggressive melanomas [27, 28]. Interestingly, using immunohistochemistry, we were able to detect increased Grm1 expression on excised tumors from mice harboring mutated BRaf^V600E^ on a PTEN null background. To assess the relationship between mutated BRaf^V600E^ and Grm1in this novel mouse model, we devised a set of *in vivo* and *in vitro* experiments aimed to elucidate molecular events that lead to Grm1 overexpression and tumorigenesis.

## RESULTS

### Activation of Grm1 expression in a mutated BRaf^V600E^ PTEN null transgenic mouse model

**Figure 1:**
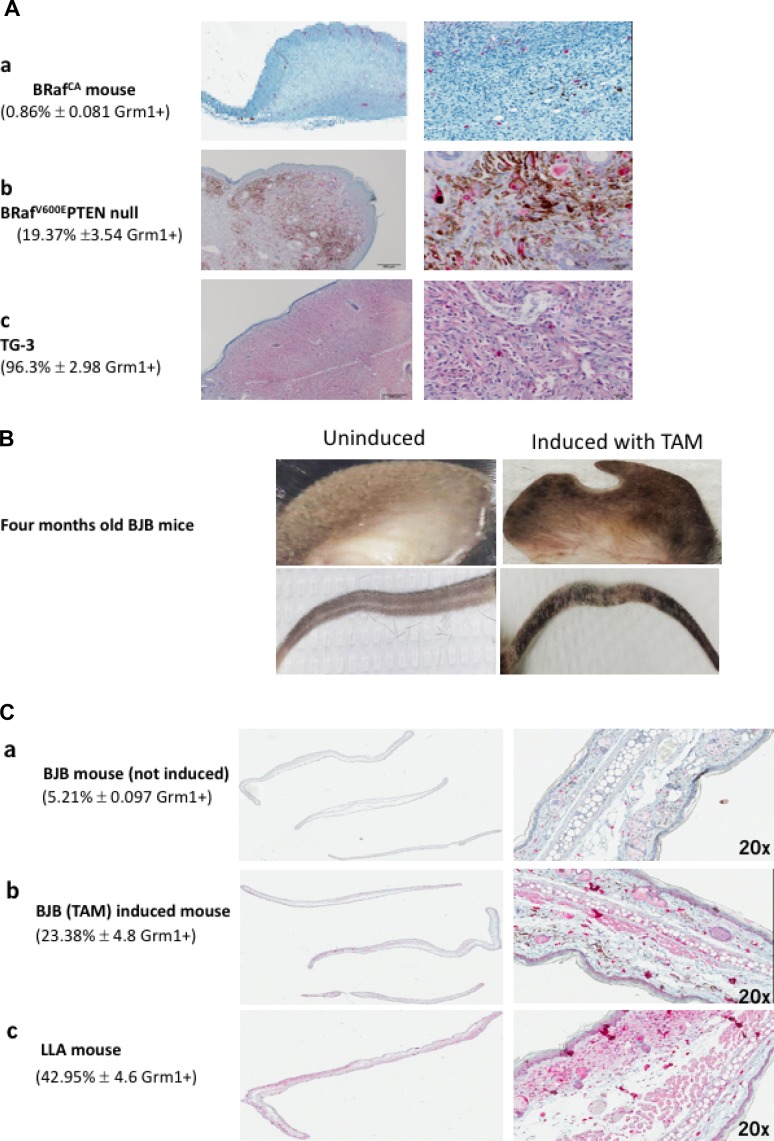
**(A)** Immunohistochemistry (IHC) with Grm1 antibody. (a) Ear sample was from BRaf^CA^ mouse (melanocytes with wild-type Braf before Cre-mediated recombination [29]). (b) BRaf^V600E^ PTEN null mouse ear (conditional melanocyte-specific tyrosinase regulated Cre recombinase and Braf^CA^, in PTEN null background induced by the inducer, 4-hydroxytamoxifen (TAM) and led to mutated BRaf^V600E^ expression only in melanocytes [27], and (c) ear from melanoma prone TG-3, with aberrant Grm1 expression with wild-type BRaf [15, 16]. The mice were 4–5 months old and the IHC were performed with 8 mice from each genotype. The percentage of positive Grm1 staining in each genotype is expressed as the average of all 8 mice ± S.D. Unbiased quantitative assessment of IHC staining is completed using a digital Aperio ScanScope GL system and Aperio ImageScope software (v 10.1.3.2028) (Aperio Technologies Inc., Vista, CA) according to the manufacturer’s protocol. (**B**) Hyperpigmentation in skin around the ears and tails only in 4 months old BJB mice induced with 4-hydroxytamoxifen (TAM). BJB mice are derived from crossing a BRaf^CA^ mouse line [loxP-BRaf(V600E)-loxP] [27] with a B6CST (Cre^ERT2^) mouse line, which harbors conditionally active Cre recombinase only in melanocytes [27]. (**C**) (a) An ear of BJB mouse that was not induced with TAM is Grm1-negative with very low Grm1 staining likely from other cell types not melanocytes. (b) Sample from an ear of BJB mouse induced with TAM show Grm1 staining, and (c) Sample from an ear of LLA (albino version of TG-3) mouse was used as positive control for Grm1 staining. IHC staining were performed with ears from five mice in each group and the percent of Grm1 positive is the average of all five mice ± S.D. Unbiased quantitative assessment of IHC staining is completed using a digital Aperio ScanScope GL system and Aperio ImageScope software (v 10.1.3.2028) (Aperio Technologies Inc., Vista, CA) according to the manufacturer’s protocol.

Activating mutations in BRaf have been detected in approximately 60% of melanoma tumors and nevi. The most common mutation, BRaf^V600E^ constitutes almost 90% of the observed mutations [25, 26]. McMahon and colleagues genetically engineered BRaf^CA^ mice that express wild-type Braf upstream of Cre-mediated recombination [29]. Subsequently the same group generated mice with conditional melanocyte-specific tyrosinase-regulated Cre recombinase and Braf^CA^. in which the presence of the inducer, 4-hydroxytamoxifen (TAM), induces mutated BRaf^V600E^ expression only in melanocytes [27]. These BRaf^V600E^ mice developed benign melanocytic hypoplasia that did not progress to tumor [27]; however if crossed with PTEN null mice, tumor development was detected with 100% penetrance [27]. Immunohistochemical (IHC) staining with Grm1 antibody on the ear tissue derived from a mouse harboring mutated BRaf^V600E^ alone (Figure 1, a panel) showed a very low percentage of positive Grm1; however, staining on the ear tissue of a mouse harboring mutated BRaf^V600E^ and null PTEN was substantially higher (Figure 1, b panel). This was compared to a control for Grm1 expression in transgenic TG-3 with aberrant Grm1 expression (Figure 1, c panel). With these unexpected observations, we set out to determine the relationship between mutated BRaf^V600E^, ectopic Grm1 expression, and loss of PTEN both *in vivo* and *in vitro*.

### BJB mouse model harbors inducible mutated BRaf^V600E^ with subsequent induction of Grm1 expression

BJB mice were produced by crossing a BRaf^CA^ mouse line [loxP-BRaf(V600E)-loxP] [27] with a B6CST (Cre^ERT2^) mouse line, which harbors conditionally active Cre recombinase only in melanocytes [27]. The application of the inducer 4-hydroxytamoxifen (TAM, 15 mg/ml) on the ears of one-month-old BJB mice for four days allows recombination of the loxP- BRaf^V600E^ -loxP in melanocytes. At eight weeks post treatment, skin hyperpigmentation around the ears and tails of BJB mice was observed as described [27] (Figure 1B). To confirm Grm 1 expression, we conducted IHC on ear samples from four-month-old, untreated and treated BJB mice (Figure 1C, panels a and b respectively). Positive Grm1 staining was detected only in TAM treated samples (Figure 1C, panel b) using an albino TG-3 as positive control (Figure 1C, panel c).

**Figure 2:**
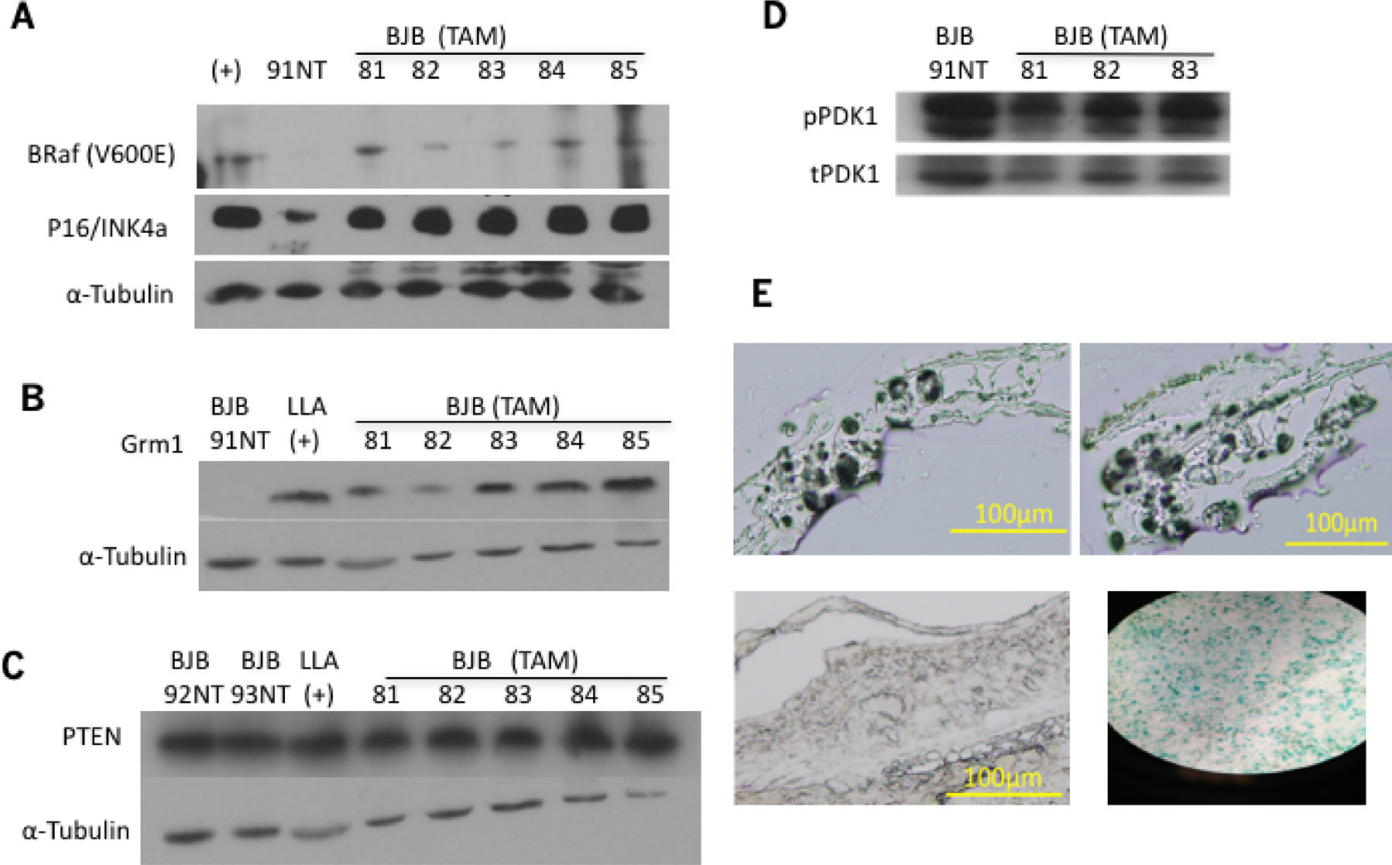
(**A**) BRaf^V600E^ expression in the ears of BJB mice was confirmed by Western immunoblot. BRaf^V600E^/PTEN null transgenic mouse used as positive control (+), BJB 91 was not treated with TAM and used as negative control. The same membrane was used to probe for p16/INK4a, and α-tubulin used as loading control. (**B**) Same set of protein lysates from (A) was used in Western immunoblots for Grm1 expression in the ears of BJB mice. LLA transgenic mouse was used as positive control, BJB 91 was not treated with TAM and used as negative control, α-tubulin used as loading control. (**C**) PTEN expression was assessed by Western immunoblot, showing preserved expression in both TAM treated and non-treated samples. α-tubulin used as loading control. (**D**) Phosphorylated PDK1 [pPDK1] expression was assessed by Western immunoblots, and demonstrated similar expression in TAM treated and untreated samples. Total PDK1 [tPDK1] was used as a loading control. (**E**) β-galactosidase staining was used to assess cell senescence using frozen sections of 14-month-old, TAM-treated BJB mouse ears. BJB TAM induced samples (top panels) show positive staining, similar to etoposide-treated MCF7 positive control (bottom right panel), while 14-month-old BJB mouse ear not induced with TAM did not show positive β-galactosidase staining (bottom left panel).

Protein from the ears of TAM treated and untreated BJB mice with BRAF^V600E^ expression only detected in TAM treated animals (Figure 2A). Additionally, levels of p16/INK4a in these samples is shown in Figure 2A. Grm1 was also expressed in the same set of samples (Figure 2B), confirming previous IHC staining (Figure 1C). We then evaluated PTEN expression in the same set of protein lysates by Western immunoblot. PTEN expression was preserved in these samples (Figure 2C). However, it was unclear if PTEN is functional in these samples. PTEN inhibits phosphorylation of PIP3, which subsequently phosphorylates PDK1 [pPDK1]. pPDK1 then phosphorylates AKT proteins (T308); thus, increased expression of pPDK1 indicates enhanced PI3K/AKT pathway activity. pPDK1 levels were very similar among untreated and TAM treated samples (Figure 2D), suggesting that PTEN is at least partially functional in these *in vivo* samples. However, because BJB mice induced with TAM never develop melanoma beyond hyperpigmentation [27], we used senescence-associated β-galactosidase staining to show that cells from TAM-treated BJB mice have undergone senescence (Figure 2E). Next, we endeavored to assess if Grm1 expression is also induced in *in vitro* cultured mutated BRaf mouse melanocytic clones.

### Stable melanocytic clones with exogenous mutated BRaf^V600E^ induce Grm1 expression

**Figure 3:**
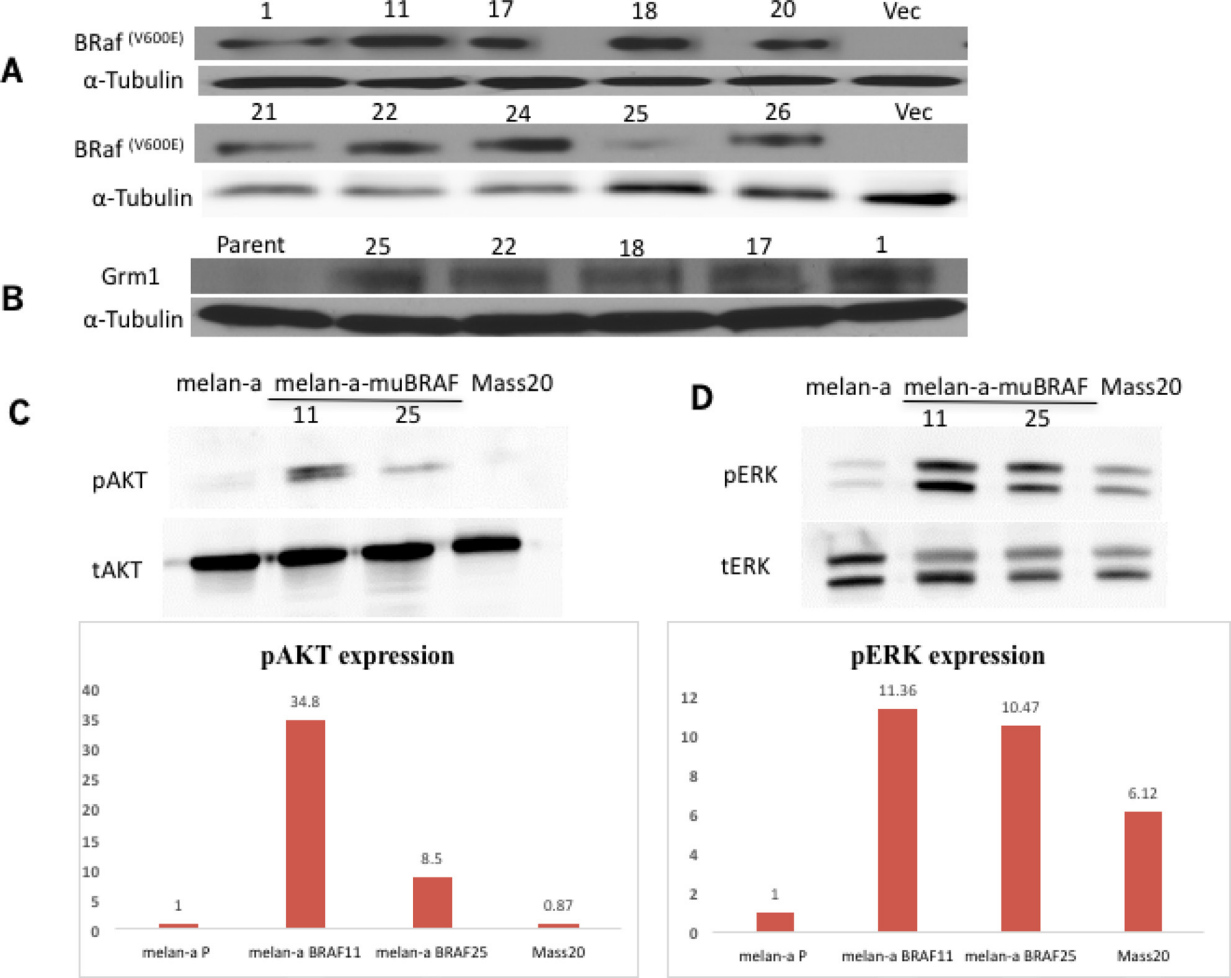
Melan-a-muBRaf clones induce Grm1 expression and downstream signaling pathways (**A**) Expression of BRaf^V600E^ in several stable melan-a-muBRaf clones was confirmed by Western immunoblot. PCIneo was used as vector control. (**B**) Several stable melan-a-muBRaf clones also demonstrated Grm1 expression by Western immunoblot. melan-a parent was used as negative control and α-tubulin as loading control. (**C**) Elevated basal levels of pAKT (T308) and (**D**) pERK1/2 were detected in two randomly selected melan-a-muBRaf clones 11 and 25 compared to the parental melan-a or MASS20 (melan-a with exogenous Grm1). Total AKT [tAKT] and total ERK1/2 [tERK] were used as loading controls. Blots were quantitated and shown as fold difference over parent control normalized to loading control.

Several stable clones of immortalized normal mouse melanocytes (melan-a) with exogenously introduced myc tagged mutated BRaf^V600E^ [melan-a-muBRAF] were isolated and mutated BRaf expression was confirmed by Western immunoblots (Figure 3A). Western immunoblots showed Grm1 expression in several representative melan-a-muBRaf stable clones but not parental melan-a cells (Figure 3B). We then selected two independent clones, 11 and 25, for further analysis. We observed higher basal levels of pAKT (Figure 3C) and pERK1/2 (Figure 3D) in both 11 and 25 clones in comparison to the parental melan-a or MASS20 (melan-a with exogenous Grm1 but maintained wild-type BRaf). MASS20 cells exhibit higher basal pERK than parental melan-a, but very low levels of pAKT *in vitro*. Elevated pAKT was detected only in aggressive *in vivo* MASS tumors [19, 20]. These data suggest that the introduction of mutated BRaf^V600E^ into normal mouse melanocytes resulted in functional BRaf, and subsequently functional Grm1, which may be able to render a malignant phenotype.

### Modulation of BRaf function or expression affects Grm1 expression in melan-a-muBRaf clones

**Figure 4:**
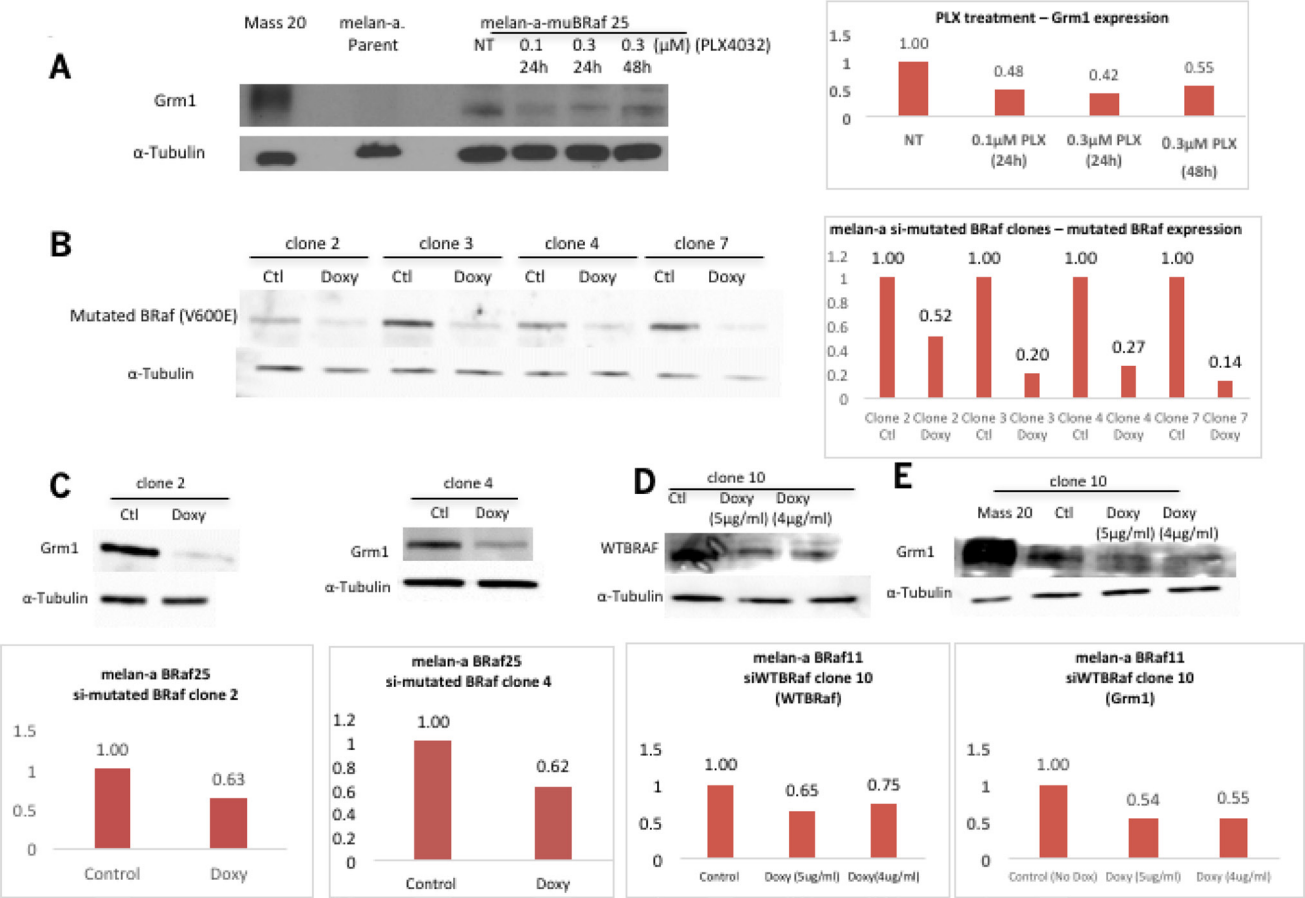
Concurrent alteration in BRaf function/expression and Grm1 expression (**A**) A reduction in Grm1 expression was detected in representative melan-a-muBRaf clone 25 when treated with a small molecule inhibitor PLX4032 for 24 hrs at 0.1 μM or 0.3 μM, or for 48 hrs at 0.3 μM. MASS20 was used as positive control for Grm1 expression, melan-a parent as negative control, and α-tubulin as loading control. Blot was quantitated as shown as a fold difference over non-treated sample normalized to loading control. (**B**) Melan-a-muBRaf (V600E) cells were transfected with Tet^R^ and siBRAF^V600E^-Tet^O^ plasmids to generate inducible melan-a siBRaf^V600E^ stable clones. Doxycycline [doxy] was applied for four days to induce siRNA expression. Western immunoblots confirmed suppression of BRAF^V600E^ induced by doxy. Control sample [ctl] not treated with doxy; α-tubulin was used as loading control. Blots were quantitated and shown as fold difference over non-treated sample normalized to loading control. (**C**) Two representative clones showed subsequent decrease in Grm1 expression with knockdown of mutated BRaf^V600E^ in Western immunoblot. Blots were quantitated and shown as fold differences over parent control normalized to α-tubulin as loading control. (**D**) Melan-a-muBRaf (V600E) clones were transfected with Tet^R^ and siWTBRaf-Tet^O^ plasmids to generate inducible melan-a siWTBRaf stable clones. Doxycycline [doxy] was applied for four days to induce siRNA activity. Western immunoblots confirmed suppression of wild-type BRaf [WTBRaf] induced by 5 μg/ml or 4 μg/ml doxy. Control sample [ctl] not treated with doxy, α-tubulin used as loading control. Blot was quantitated and shown as fold differences over parent control normalized to loading control. (**E**) Silencing of WTBRaf led to a similar degree of down-regulation of Grm1 expression compared to MASS20 positive control. α-tubulin used as loading control. Blots were quantitated and shown as fold differences over parent control normalized to loading control.

We examined the relationship between BRAF^V600E^ and Grm1 by modulating mutated BRAF^V600E^ function with a specific inhibitor, PLX4032, or expression with silencing RNA [siRNA]. Treatment of melan-a-muBRaf clones with PLX4032 for 24 hours at 0.1 μM or 0.3 μM, or 48 hours at 0.3 μM led to decreased Grm1 expression in either a dose- or time-dependent manner, compared to untreated control (Figure 4A).

To manipulated mutated vs. wild-type BRaf expression, we used an inducible TetR-siRNA system. Mutated BRaf^V600E^ protein levels were evaluated following incubation with the inducer doxycycline [doxy] for 4 or 6 days in several isolated clones. Substantial reduction in mutated BRaf protein levels was detected in all four doxy-treated clones (Figure 4B). We then selected two clones (clones 2 and 4) to assess Grm1 expression and showed decreased Grm1 expression in cells with knockdown of mutated BRAF^V600E^ expression (Figure 4C).

In parallel, we also produced an inducible cell line with siRNA to wild-type BRaf [wtBRaf]. We were surprised to see that a reduction in wtBRAF expression (Figure 4D) also led to a corresponding reduction of Grm1 expression (Figure 4E). Taken together, these results suggest that a decrease in the function or expression of either mutated or wild-type BRaf led to a reduction in Grm1 expression, suggesting that homo- or hetero-dimerization of BRaf is required for its kinase activity [30] and regulation of Grm1 expression.

### PTEN functionality may be altered in melan-a-muBRaf clones

**Figure 5:**
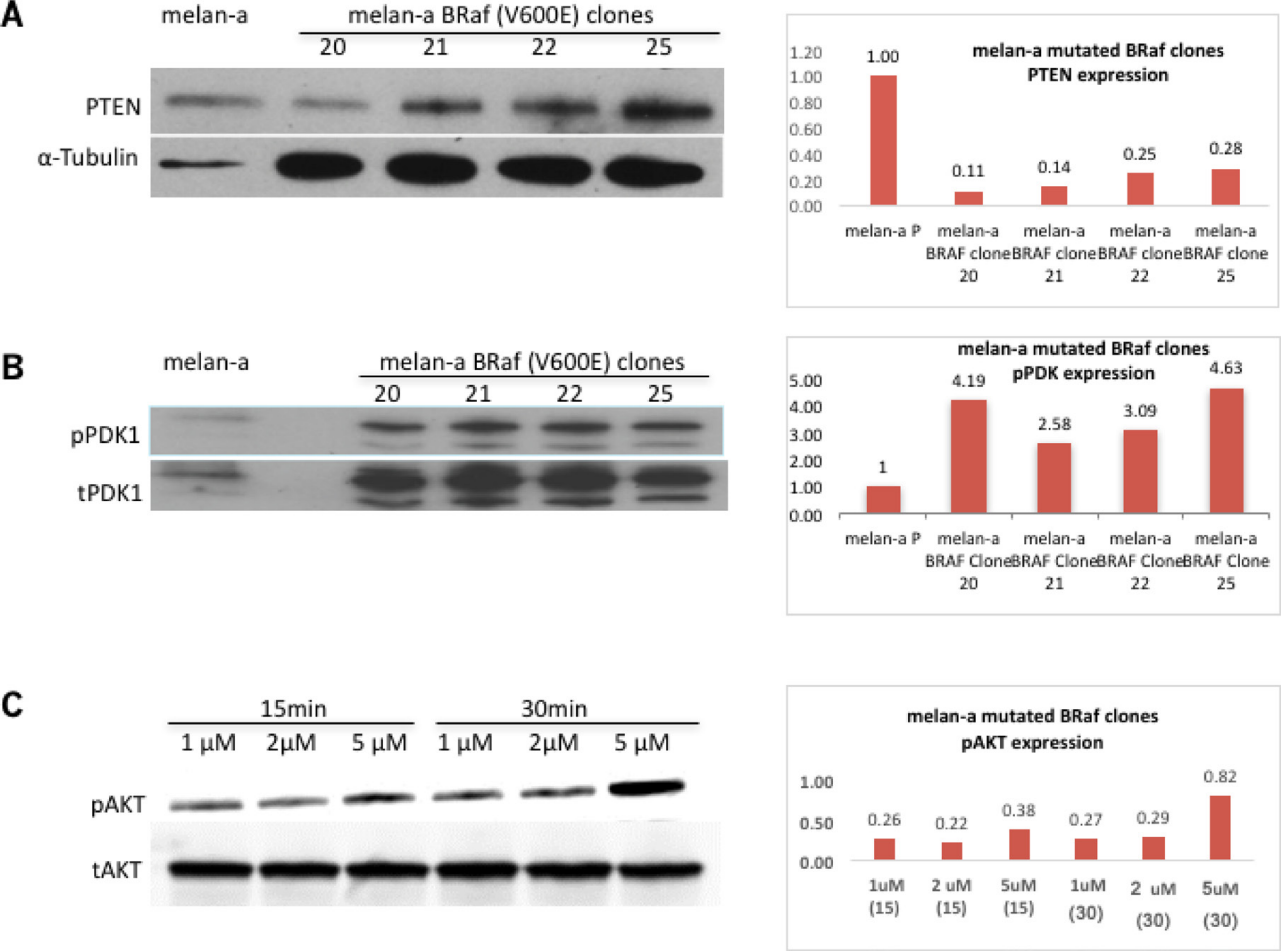
PTEN functionality may be altered in melan-a-muBRaf clones (**A**) Western immunoblot showed PTEN expression in stable melan-a mutated BRaf^V600E^ clones that are Grm1-positive, melan-a parent was used as control and α-tubulin as loading control. Blots were quantitated and shown as fold difference over parent control normalized to loading control. (**B**) Western immunoblot shows expression of pPDK1 in melan-a mutated BRaf^V600E^ clones and very little in melan-a parent. Membrane was stripped and re-probed with total PDK1 as loading control. Blots were quantitated and shown as fold difference over parent control normalized to loading control. (**C**) Five different melan-a mutated BRaf^V600E^ clones treated with increasing concentrations of PTEN inhibitor bpV (phen) (1 μM, 2 μM and 5 μM) for 15 and 30 minutes showed pAKT levels increasing in a dose dependent manner. Membrane was stripped and re-probed with total AKT as loading control. An example of melan-a-muBRaf clone-22 is shown. Blots were quantitated and shown as fold difference over loading control.

We previously showed that PTEN expression levels *in vivo* are not affected in BJB mice (Figure 2C). Its functionality, as evaluated by pPDK1 levels, remained stable (Figure 2D), suggesting that PTEN is unaltered by mutated BRaf *in vivo*. However, activated pAKT was detected in melan-a-muBRaf clones (Figure 3C), indicating possible PTEN loss of function or dysfunction in these cells, given that PTEN is a negative regulator of PI3K/AKT. In the absence of functional PTEN, phosphatidylinositol (3,4,5)-triphosphate (PIP3) is not dephosphorylated, which subsequently phosphorylates PDK1. pPDK1 then phosphorylates AKT proteins (T308). PTEN and pPDK1 were detected in all four melan-a-muBRaf clones (Figure 5A and 5B). We then treated these cells with increasing concentrations of an inhibitor bpV (phen) (1 μM, 2 μM and 5 μM) for 15 and 30 minutes. pAKT levels increased in a dose- and time-dependent manner, suggesting that PTEN in melan-a-muBRaf cells is at least partially functional and was responsive to its inhibitor bpV (phen) (Figure 5C).

### Grm1 induced by mutated BRaf is functional

**Figure 6:**
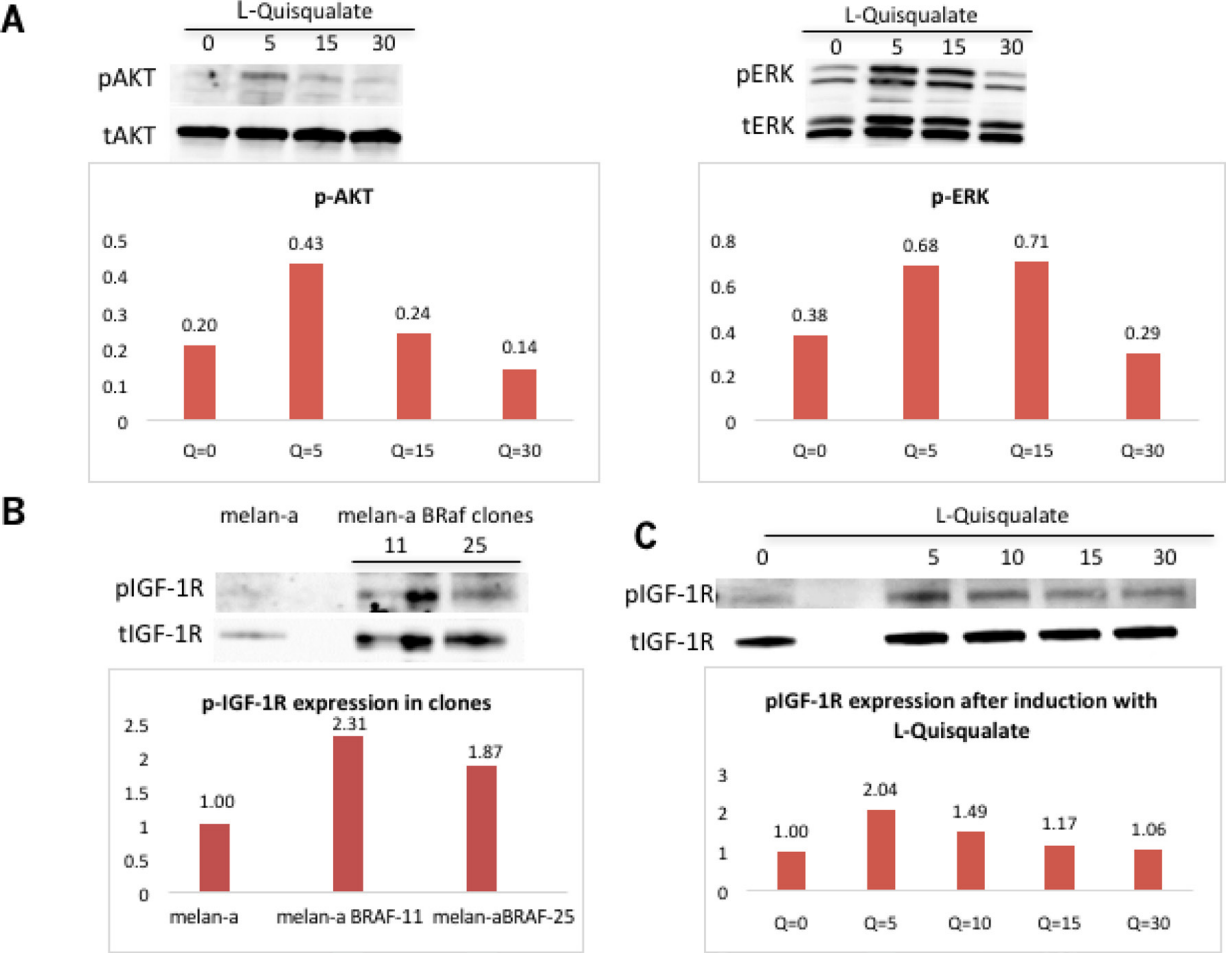
Grm1 induced by mutated BRaf is functional (**A**) L-quisqualate [Q] (10 μM) used to stimulate a representative melan-a-muBRaf clone led to increased levels of downstream pAKT and pERK1/2. Membrane was stripped and re-probed with total AKT or total ERK as loading controls. Blots were quantitated and shown as fold differences over loading control. (**B**) Western immunoblot showed expression of phosphorylated IGF-1R [pIGF-1R] is expressed in melan-a-muBRaf clones, but not in melan-a parent. Membrane was stripped and re-probed with total IGF-1R as loading control. Blots were quantitated and shown as fold difference over parent control normalized to loading control. (**C**) Melan-a-muBRaf was treated with Grm1 agonist, Q, for up to 30 min. pIGF-1R levels were modulated by Grm1 stimulator. The membranes were stripped and re-probed with total IGF-1R as loading control; blots were quantitated and shown as fold differences over treated sample at time 0 normalized to loading control.

To test if the Grm1 expression induced in melan-a-muBRaf clones is functional, we conducted induction experiments using the Grm1 agonoist, L-quisqualate [Q] (10 μM). Western blots were performed to analyze the levels of pERK and pAKT, as described previously [19, 20]. We showed that both pAKT and pERK1/2 were upregulated by Q treatment with the maximum level at 5-15min, which gradually returned to basal levels by 30min, consistent with our earlier results (Figure 6A) [19, 20].

### Activation of PI3K/AKT pathway downstream of Grm1 induced by mutated BRaf^V600E^ is mediated through transactivation of IGF-1R via Src

**Figure 7:**
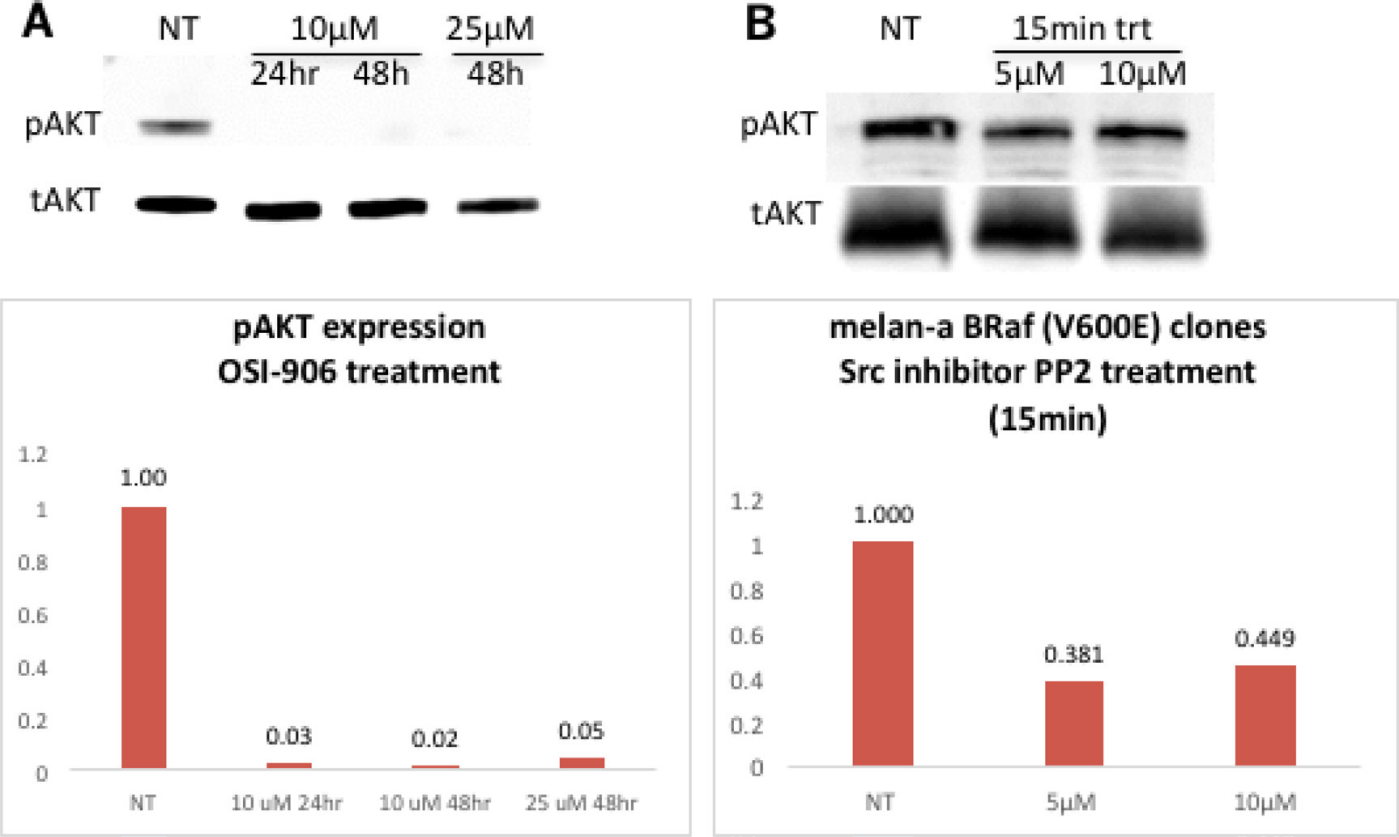
Activation of PI3K/AKT pathway downstream of Grm1 induced by mutated BRAF^V600E^ is mediated through IGF-1R via Src (**A**) Western immunoblot shows knockdown of pAKT expression in a representative melan-a-muBRaf clone 11 after treatment with IGF-1R inhibitor OSI-906 at increasing concentrations (10 μM or 2 5 μM) for 24 or 48 hours. Membrane was stripped and re-probed with total AKT as loading control; blots ere quantitated and shown as fold differences over non-treated control normalized to loading control. We performed this study with three different mutated BRaf clones (clone 11, 20 and 25) treated at three independent times. (**B**) Western immunoblot shows a representative melan-a-muBRAF clone 25 treated with an Src inhibitor, PP2 at 5 μM or 10 μM for 15 minutes, with subsequently lower expression of pAKT. The membrane was stripped and re-probed with total AKT as loading control; blots were quantitated and shown as fold differences over non-treated control normalized to loading control. Three independent melan-a-muBRaf clones were used (clones 11, 21 and 25) and the study was performed at three different times.

The insulin-like growth factor-1 [IGF-1] is an activator of the AKT pathway. Previous studies in our lab found that Grm1 modulates phosphorylation of AKT via IGF-1R transduction in Grm1-mouse melanocytes (MASS clones) [21]. We demonstrated that IGF-1R is phosphorylated in melan-a-muBRaf clones, but not in the parental melan-a cells with wild-type BRaf (Figure 6B). Furthermore, we also demonstrated that increased modulation of pIGF-1R can be achieved using Grm1 agonist, Q (Figure 6C). We then treated melan-a-muBRaf cell lines with different concentrations (10 μM or 25 μM) of IGF-1R inhibitor, OSI-906, to examine if activation of the PI3K/AKT pathway was indeed mediated through IGF-1R. After treatment with OSI-906 for 24 and 48 hours, pAKT expression was completely knocked down, indicating activation of the AKT pathway in these cells was mediated through IGF-1R as shown in our earlier studies [21]; a representative of melan-a-muBRaf clone 11 is shown in Figure 7A.

We previously showed that Src family tyrosine kinases serve as intermediates between Grm1 and IGF-1R, as also suggested by others [21]. In the current study, we tested if treatment with an Src inhibitor, PP2, could modulate pAKT. Protein lysates from melan-a-muBRaf cell lines treated with PP2 at 5 μM or 10 μM for 15 minutes had lower levels of pAKT compared to untreated control (Figure 7B). Taken together, these results support our previous observations of Grm1 modulation of the PI3K/AKT pathway through crosstalk with IGF-1R via Src [21].

### BRaf^V600E^ upregulated p15 expression through TGFβ signaling in BRaf^V600E^ mice but not TG-3 mice with wild-type BRaf

**Figure 8:**
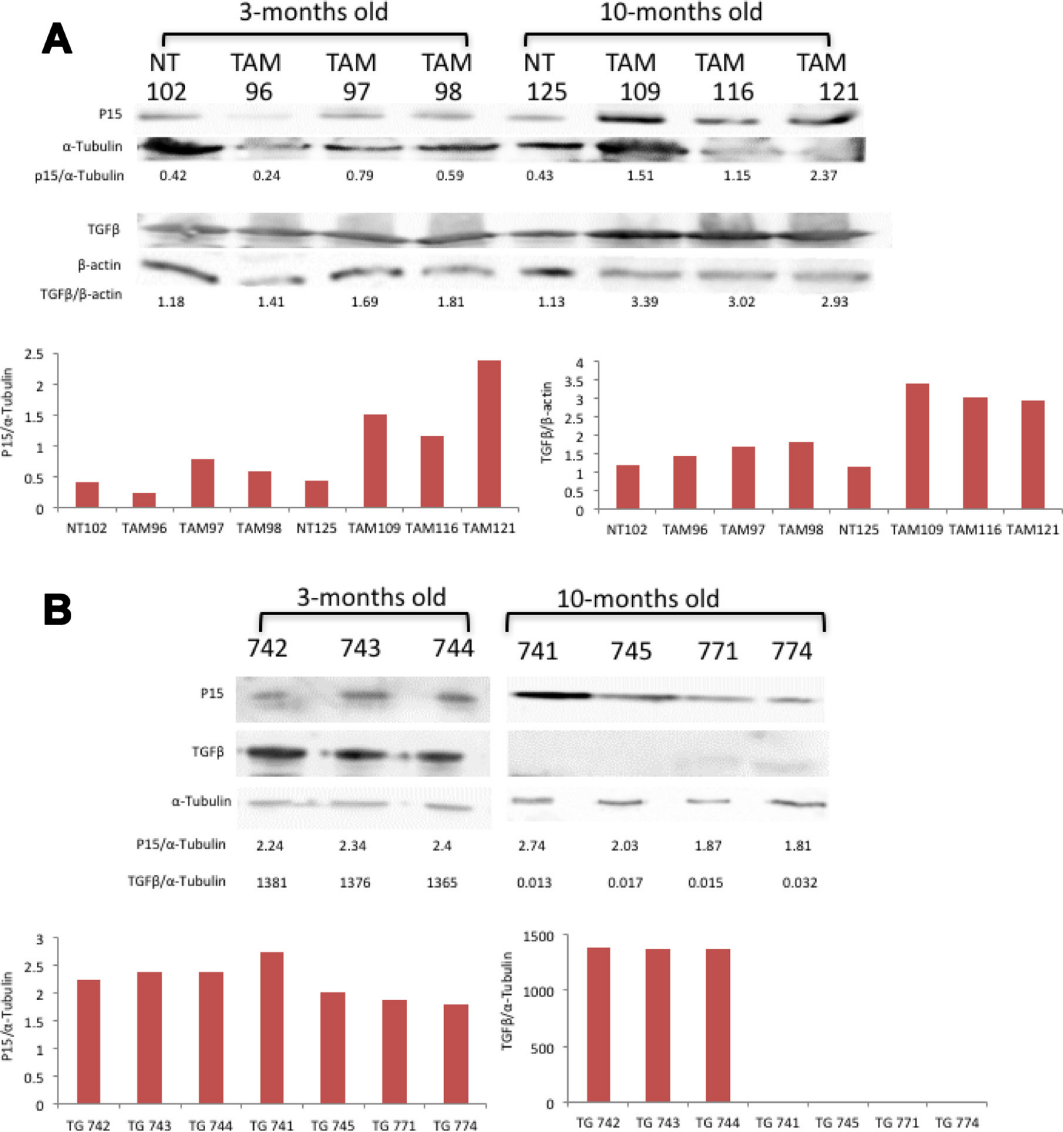
**(A)** TGFβ induced p15 in mutated BRaf but not wild-type BRaf mice. (A) Western immunoblots on ear protein lysates prepared from three-or ten-month old BJB mice, either not treated (NT) or treated with 4-hydroxytamoxifen (TAM, 15 mg/ml) and probed with antibodies to p15, α-tubulin, TGFβ and β-actin. **(B)** Western immunoblots on ear protein lysates prepared from three- or ten-month old TG-3 mice, and probed with antibodies to p15, α-tubulin, and TGFβ. All blots were quantitated and shown as fold differences normalized to loading control.

The results presented thus far, that the introduction of mutated BRaf^**V600E**^ can induce expression of functional Grm1, would indicate that a malignant phenotype much like TG-3 should result from a BRaf mutation *in vivo* if Grm1 is also present. However, in BJB mice, as previously described, only melanocytic hyperplasia is observed, despite expression of functional Grm1. We predicted that cells that temporally express mutated BRaf^**V600E**^ prior to Grm1 continue to undergo senescence. Deletion of the entire CDKN2B-CDKN2A gene cluster is one of the most common genetic events in cancer. Loss of CDKN2A-encoded p16 leads to tumor promoting growth properties; in contrast, the consequence of loss of CDKN2B-encoded p15 and its relationship with tumorigenesis is less clear. Recently, Ridky and co-workers demonstrated that activation of p15 in mouse melanocytes by mutated BRaf^V600E^ via TGFβ led to growth arrest of the cells [31]. We were interested to know if similar regulation occurs in BJB mice. We took protein lysates from young and old BJB mice and assayed for p15 and TGFβ protein levels (Figure 8A). At three-months of age, p15 levels were similar among untreated (wild-type BRaf) and TAM-treated (mutated BRAF^V600E^) BJB mice. In contrast, in ten-month old animals, p15 protein levels were much higher in TAM-treated mutated BRAF^V600E^ mice compared to untreated mice with wild-type BRaf (Figure 8A). Parallel to higher p15 levels in older BJB mice, TGFβ expression was also upregulated (Figure 8A). Similar analyses were performed in young and old TG-3. TG-3 mice spontaneously develop metastatic melanoma with 100% penetrance [15]. Regardless of the age of TG-3 mice, p15 protein levels were very similar (Figure 8B). Surprisingly, younger TG-3 exhibited very high TGFβ levels while in older TG-3 mice TGFβ was barely detectable (Figure 8B). Taken together, these results further confirm that upregulation of p15 via elevated TGFβ by mutated BRaf led to cell growth arrest, and no tumor formation until additional genetic aberration such as ablation of PTEN. On the contrary, TG-3 mice harbor wild-type BRaf; therefore, without the induction of TGFβ or p15 expression, cells do not undergo growth arrest, thus resulting in continual, consistently predictable tumor formation.

## DISCUSSION

Previously, we have shown that ectopic expression of Grm1 alone in melanocytes was sufficient to induce spontaneous melanoma development with 100% penetrance in transgenic mouse models [15, 16]. We also showed that introduction of exogenous Grm1 into immortalized mouse melanocytes, mammary epithelial cells, or kidney epithelial cells led to a modest *in vitro* transformed phenotype, and robust *in vivo* tumorigenesis [19, 32, 33]. The human relevance of these results was revealed not only in melanoma, but also in breast and renal cell carcinoma, suggesting that Grm1 is a player in numerous malignancies [34–37]. How Grm1 expression becomes dysregulated in melanocytes remains interesting but unknown. The current study began when we detected Grm1 expression in a transgenic mouse model of melanoma harboring mutated BRaf^V600E^ in a PTEN null background.

Although the oncogenic expression of mutated BRaf or NRas elicits anomalous melanocyte proliferation, they are insufficient to induce melanoma development. Instead the cells undergo oncogene-induced senescence, resulting in the formation of benign nevi only [27, 38, 39]. Various groups have demonstrated that mutated BRaf or NRas accompanied by ablation of coding sequences or loss of function mutations in either PTEN or p16/INK4 will stimulate senescent cells to re-enter the cell cycle and progress to melanoma *in vivo* [27, 40, 41]. It has been postulated that oncogene overexpression within the MAPK pathway may upregulate p16 and p53, leading to oncogene-induced senescence [38, 39].

The INK4a/ARF locus produces two unique proteins, p16/INK4A and p14/p19ARF. p16/INK4A directly inhibits the cyclin D-dependent kinases CDK4/6 and prevents it from phosphorylating Rb, which is critical for progression into S phase. p14/p19ARF binds to MDM2 and stabilizes p53, thus arresting cells in G1 or G2 phase [42]. Allelic loss of the INK4a/ARF locus has been observed often in metastatic human melanoma cell lines as well as in familial melanoma. In cultured immortalized melanocytes, it has been shown previously by other groups and confirmed by our lab that melan-a cells are null for p16/INK4a [43]. That Grm1 expression is induced by mutated BRaf in melan-a cells suggests that p16/INK4a is probably not involved. In addition, p16/INK4a was also detected in BJB mouse ears regardless of treatment with TAM, again suggesting that p16/INK4a is probably not involved in the activation of Grm1 expression *in vivo*. Ridky and colleagues demonstrated that increased levels of TGFβ-induced p15 mediate the growth arrest of cells carrying mutated BRaf. This is likely why mutated BRaf alone was not sufficient to induce tumorigenesis without additional genetic aberrations. Unlike mutated BRaf, aberrant Grm1 expression in melanocytes is sufficient to induce cell transformation *in vitro* and robust tumor formation *in vivo* with no alteration in p15 expression and almost undetectable TGFβ. How TGFβ is regulated in Grm1 positive TG-3 is not known and is currently being investigated.

We postulate that Grm1 may be an intermediate signaling molecule in BRAF^V600E^ expressing melanomas that contributes to melanomagenesis. Our melanoma-prone Grm1 transgenic mouse models (TG-3 and EPv) do not harbor a BRAF^V600E^ mutation (unpublished data), which is consistent with our *in vitro* cultured cell studies, that introduction of exogenous Grm1 cDNA into melan-a cells (MASS clones) did not induce mutation of BRaf. In melan-a-muBRaf clones, high basal pAKT levels are detected and increased even more when stimulated with Grm1 agonist Q. In contrast, in cultured Grm1-melan-a, MASS cells, basal pAKT were barely detectable; however, when the cells were stimulated with Grm1 agonist, L-Quisqualate, a dose-dependent increase in pAKT was detected [19, 20]. Furthermore, in excised *in vivo* allografted MASS tumors, elevated pAKT was observed compared to its cultured counterpart [19, 20], suggesting that in the *in vivo* microenvironment, the PI3K/AKT pathway was activated.

The BRaf monomer contains an N-lobe of five antiparallel-strands, a regulatory C helix, and a C-lobe with a key loop called the activation segment. Active BRaf kinase maintains a closed conformation by forming a dimer. Several modifications might change the stability of the closed state, such as phosphorylation of the activation segment or protein-protein interactions. Inward movement of the C helix and the in-position movement of a Phe residue on the Asp-Phe-Gly (DFG) motif located on the activation segment may be crucial for protein catalysis. In active kinases, the DFG motif adopts an “in” conformation and an Asp residue is oriented toward bounded ATP, whereas the conformation of the DFG motif is flipped outward in inactive kinase [44, 45]. Evidence shows that Arg509 located on the C-terminal tip of the C helix is the interface for side-to-side dimerization [46]. It is believed that BRaf dimerization is the result of allosteric coupling with conformational changes of the activation segment [30]. It was shown that mutated BRAF^V600E^ is a kinase-impaired BRaf mutant, but has an intact dimerization interface and transactivation activity. It also has the ability to remain activated because it has increased dimerization potential and can activate the MAPK pathway without ligand binding. Vemurafenib (PLX4032) was a promising specific BRaf^V600E^ small molecule inhibitor [9]. In the first six to eight months of treatment, patients showed recovery and control of tumorigenesis. However, continuing on this inhibitor caused reactivation of the MAPK pathway due to wild-type BRaf dimerizing with other RAF family proteins such as KSR and CRAF [47–50]. BRaf was recruited to the membrane by GTP-RAS, and then dimerized with CRAF, followed by conformational change. CRAF was transactivated through BRaf and phosphorylated MEK. Therefore, even if the mutant BRAF^V600E^ was inhibited by Vemurafenib/PLX4032, melanoma cells still could activate the MAPK pathway through wild-type BRaf [9]. When we conditionally altered either mutated or wild-type BRaf expression by inducible silencing RNA that modulated homo- or hetero-dimer formation, a parallel reduction in Grm1 expression was also detected. These results suggest that either homo- or hetero-dimer formation by BRaf is one of the critical regulators in Grm1 expression.

PTEN expression is present in melan-a-muBRaf clones, but its functionality is questionable given the upregulated pPDK1 and pAKT, which PTEN normally inhibits (Figure 5). Furthermore, we also detected a dose-dependent increased in pAKT in the presence of PTEN inhibitor, suggesting that PTEN is still at least partially functional. Our earlier studies identified that AKT2 was the predominant isoform that was activated by Grm1 in a PTEN independent fashion [20]. Grm1 mediates activation of AKT2 via Src to transactivate IGF-1R [21]. Our current data in this study support similar mechanisms.

Finally, we confirmed that the activation of p15 and TGFβ drive aged BJB mice into cell senescence and no tumor formation, while in aged TG-3 mice the increased in p15 and TGFβ was not observed and actively growing melanocytes lead to tumor formation. Taken altogether, the results of our current study suggest that expression of mutated BRaf^V600E^ can induce Grm1 expression in melanocytes, but is not sufficient to induce spontaneous melanoma formation. In contrast if Grm1 expression is turned on first, it is sufficient to yield a full tumorigenic phenotype *in vivo* in the absence of mutated BRaf. This temporal regulation of Grm1 and mutated BRaf is critical in determining if a cell will enter oncogene-induced senescence or tumorigenesis. We suspect that if mutated BRaf is expressed first, then a critical but unknown pathway is also activated, preventing Grm1 -induced neoplasia.

## MATERIALS AND METHODS

### Cell culture

Immortalized normal mouse melanocytes (melan-a cells) were provided by Dr. Dorothy Bennett (St. George’s University of London, UK) and grown in RPMI-1640 medium (Sigma, St. Louis MO) with 10% fetal bovine serum (FBS) (Sigma, St. Louis MO), 100 U/ml of penicillin/streptomycin (Gibco, Grand Island, NY), and 200 nM 12-O-tetradecanoyphorbol-13-acetate [TPA] (Sigma, St. Louis MO). Melan-a BRaf clones were grown in the selection medium contains 10% FBS, 100U/ml penicillin/streptomycin, and G418 (200 ug/ml).

### Plasmid purification

Plasmid purification was performed for TetR, siMUBRaf, and siWTBRaf. QIAprep Spin Miniprep Kit was used to prepare purified plasmid per manufacturer’s protocol. Concentration of each plasmid DNA was determined by DNA agarose gel electrophoresis using 0.8% agarose gel, and compared to known concentration of DNA marker, λ-Hind III. Purified plasmid DNA was then precipitated with 250 mM ammonium acetate and 100% cold ethanol.

### DNA transfection

DNA transfections were performed using DOTAP transfection reagent (Roche, Basel, Switzerland) following manufacturer’s instructions. pEFmycB-Raf (V600E) plasmid was provided by Dr. Richard Morales (Sheridan *et al*., 2008). The plasmid was co-transfected with geneticin [G418] resistance plasmid PCIneo used for drug selection of stable clones. The PCIneo plasmid alone (4 μg) for vector control. Stable clones were selected by resistance to G418 (200 μg/ml) (Sigma, St. Louis, MO). Tet-R plasmid (4 ug) was co-transfected with pTinRNA mutated BRaf (V600E) plasmid (4 ug) or pTinRNA wild-type BRaf plasmid (4 ug) into melan-a BRaf clones. Stable clones with silencing mutated BRaf (V600E) were selected by resistance to G418 (200 ug/ml) and hygromycin (0.25 ug/ml), while stable clones with silencing wild-type BRaf were selected by resistance to G418 (200 ug/ml) and hygromycin (0.01 ug/ml).

### Immunochemical staining

Fresh tissue samples were fixed in formalin then transferred to Rutgers Cancer Institute Biospecimen core for staining with Grm1 antibody followed by Discovery RedMap Kit (Ventana Medical Systems, Inc. Tucson, AZ), positive stained cells will appear red.

### BJB and TG-3 mouse strains

The transgenic mouse model BJB, was generated by breeding a Braf^CA^ mouse (loxP-Braf(V600E)-loxP) which encodes a germline conditional Braf (V600E) allele, with a B6CST mouse (CreERT2) which harbors conditionally active CreERT2 only in melanocytes [29, 51]. In order to induce the rearrangement of the loxP-Braf(V600E)-loxP locus in melanocytes of BJB mice, 15 mg/ml of 4-hydroxytamoxifen (Sigma, St. Louis, MO) was applied directly to the ears of one-month old mice for 4 days. BJB mice were sacrificed at various times after 4-hydroxytamoxifen treatments. Protein was extracted from the ears and used to perform Western immunoblots as described [32]. TG-3 mice were from our own stock.

### Functionality assay of grm1

Cells were grown in 100-mm plates incubated with growth medium, which is glutamine/glutamate free supplemented with glutamax (Gibco) plus 10% dialyzed serum for 3 days. Then cells were split to 60-mm plates with approximately 4 × 10^5^ cells per plate. After cells were attached to plates, we replaced the medium to without serum, the following day the cells were treated with Grm1 agonist, quisqualate (Q, 10uM), for various time points, protein lysates prepared and performed western immunoblot for protein analysis.

### Treatment with inhibitors

Cells were plated at 2 × 10^5^/60 mm once the cells are attached, we changed the growth medium to serum free RPMI-1640 medium. The next day, the cells were treated with inhibitors, including mutated BRaf inhibitor (PLX 4032, Plessikon, Inc), PTEN inhibitor (bpV(phen), Abcam], IGF-1R inhibitor (OSI-906, OSI Pharmaceuticals Inc) and Src inhibitor (PP2, Calbiochem). The protein lysate was prepared for Western immunoblots.

### Senescence-associated β-galactosidase staining

Frozen tissue sections from BJB mouse ears were embedded in 500 ul of OCT compound for 15 minutes at room temperature. Samples were then treated with 0.3% potassium permanganate (Sigma) for 30 minutes. Two washes with PBS then treated with 0.5% oxalic acid (Sigma) for 12 minutes before undergoing another two washes with PBS. β-galactosidase staining was performed following the manufacturer’s instruction (Cell Signaling).

### Antibodies

Total-AKT, phospho-AKT (Thr308), total-ERK1/2, phospho ERK1/2, total PDK1, phospho-PDK1, Phospho-IGF-I Receptor β (Tyr1135/1136)/Insulin Receptor β (Tyr1150/1151) (19H7), total IGFR, PTEN and BRaf (55C6) were purchased from Cell Signaling Technology (Danvers, MA); Anti-p16INK4a antibody was purchased from Neo Markers (Fermont, CA); anti-mutated BRaf (V600E) antibody was purchased from New East Biosciences (Malvern, PA); anti-Grm1 antibody was purchased from R&D Systems (Minneapolis, MN); anti-p15 was from Santa Cruz; anti-β actin was from ThermoFisher; monoclonal -Tubulin antibody was obtained from Sigma (St. Louis, MO); anti- rabbit secondary was purchased from Merck ; anti-mouse secondary was purchased from Sigma (St. Louis, MO). Anti-sheep secondary was purchased from R&D Systems (Minneapolis, MN).
